# A Colonial Congress

**Published:** 1889-03-02

**Authors:** 


### A COLONIAL CONGRESS.

THE Intercolonial Medical Congress of Australasia met on January 7th at the Melbourne University. At the inaugural address delivered by his Excellency the Governor in the Wilson Hall, there were over 500 gentlemen present, many of them members of the medical profession, who practise in different colonies or in the United Kingdom. The dicta of foreign medical authorities is often disregarded by Australian practitioners, hence we hope valuable results from this Congress, at which the reasons of these departures from established methods are to be stated and discussed. Humanity is subject to diseases in Australia which do not assail dwellers in Europe, and the interchange of ideas among capable men cannot fail to be beneficial. The opening address was given by Mr. T. N. Fitzgerald (president), and in it he touched on various questions which would be brought forward for discussion, and, he hoped, solution. There was the presence of melancholia amongst their population, which, considering the busy life led by most people, was strange. Then statistics show that there are more deaths from drink in Australia than in England, and it appeared that the climate of the colonies made the use of alcohol more potent. Then the prevalence of phthisis and typhoid could probably be proved to result from defective drainage. The youth of Australia Mr. Fitzgerald regarded as healthy and vigorous. For his part he thought it better that there should be football accidents, than that their young men should grow up imbecile and dyspeptic. All this was merely skirting on the outside of the work which lay before the congress, and serious business began the following day, with Dr. Stirling's address on "Surgery as a Science." Surgery, he said, was no longer merely an art; it had become a science. But while as an art the craft was within a measurable distance of finality, as a science it was only in a healthy and vigorous infancy, but afforded abundant hopes of a splendid maturity. As an art, surgery, as well as medicine, long remained a calling apart; but as a science, its progress soon became inseparably connected with that of physiology or biology. Modern surgery had accomplished remarkable achievements in the surgery of the brain and spinal cord. Based upon a large variety of facts, drawn partly from accurate pathological observations, partly upon a series of oft-repeated experiments of the biological laboratory, certain deductions were drawn which enabled the surgeon to calculate, with a truly scientific precision, the locality of certain lesions. Dr. Stirling's address was delivered with considerable elocutionary power, and was heartily applauded. Dr. P. Smith read a paper on dipsomania, and submitted a powerful argument in favour of the establishment of buildings for the treatment of persons enslaved by drink. Intoxication, he said, was the outward expression of an internal morbid state, and not in itself a disease, and the originating causes of inebriety were so analogous to those of insanity that if the one was a physical disease so was the other. The essential conditions of successful treatment were absolute abstention from alcohol, and a sufficient time for the reparation of the diseased tissue.

On January 9th a most interesting discussion on hydatid disease took place. Dr. W. Gardner (Adelaide) read a paper on "The Surgical Treatment of Hydatids," dividing the subject into three parts, the first describing all the various surgical methods employed in the treatment of hydatids, the second consisting of statistics showing the results of the various methods of treatment, and the third explaining his own views and methods, and the results he had obtained during the last four years. The methods adopted were (1) aspiration or tapping with fine trocar and canula, with or without the injection into the cyst of iodine bile, etc. (2) Electrolysis. (3) Introduction and retention of large trocar and canula, of which there were four subdivisions. (4) Abdominal and thoracic section, allowing the membranes to be expelled spontaneously, of which method there were two subdivisions—(a) the insertion of Chassaignac's rubber tube, with immediate stitching of the cyst wall to the abdominal walls in the abdominal cases; (b) the same treatment, with a preliminary operation. (5) Abdominal and thoracic section associated with the immediate removal of the whole or the bulk of the cysts by douching with boric acid lotion, and the use of long holding forceps, and finally the insertion of a rubber tube. The results of the various methods of treatment gave the following percentages:—

| | Cases. | | Deaths. | | Percentage of Deaths. |
|---|---|---|---|---|---|
| Aspiration | 411 | ... | 73 | ... | 17·76 |
| Electrolysis | 13 | ... | 1 | ... | 7·69 |
| Caustic | 84 | ... | 25 | ... | 29·76 |
| Radical operations | 46 | ... | 8 | ... | 17·39 |
| Section (Adelaide) | 35 | ... | 6 | ... | 17·14 |
| Retained canula | 90 | ... | 24 | ... | 26·63 |
| Sections thoracic | 9 | ... | — | ... | — |
| Do. abdominal | 23 | ... | 2 | ... | 6·25 |

Dr. Thomas, of Adelaide, said he was convinced that patients suffering from pulmonary hydatids were most safely treated by free openings, but he quite disagreed with Dr. Gardner by strongly condemning injections after operations. Dr. Sydney Jones said he was not inclined to give up aspiration *in toto*, but he discarded the retained canula, caustics, and injections. Mr. T. N. Fitzgerald said that for more than 25 years he had adopted the puncture or tapping treatment in cases of hydatid disease with great success. Only in a few instances did he use aspiration, and he had known it to cause death by extravasation of blood through tearing away the tissue. In the whole course of his experience he only remembered one case of death from tapping, and that was a woman who died of acute inflammation of the liver. From one young fellow he took eight pints of fluid, and the patient was in the saddle hunting fourteen days after the operation, and was never troubled with hydatids again. The only case of hydatids in the brain which had come under his notice was that of a railway porter, who had partial paralysis. He tapped the patient through the ear, and took from 6 oz. to 8 oz. of fluid out of his brain, and the man was perfectly cured of his paralysis. Dr. B. Smith (Melbourne) advocated the desiccating or deolysing method of treating hydatid in the lungs. He deprived the patient of all fluid for 24 hours, except the small quantity given with the medicine, and for four or five days limited him to half a-pint daily, and he also administered half a tea-spoonful of common salt to increase the density of the blood, and thereby promote the desiccation of the cyst. In one case the cyst was expectorated four weeks after treatment, and in another one week after.

A long discussion on lunacy legislation took place after lunch, this being a subject much to the fore in Australia just now. His Excellency and Lady Loch gave a large reception on Tuesday evening in honour of the congress, and altogether Melbourne society is either medical or nothing just now, and conversation is confined to the cheerful subject of disease.

*(To be continued.)*

### HOSPITAL SATURDAY FUND.

THE first meeting for the present year of the delegates of the Hospital Saturday Fund was held at the Board-room, 41, Fleet Street, on Saturday last, under the presidency of Mr. H. N. Hamilton-Hoare (hon. treasurer), when the credentials of about one hundred and forty representative delegates were verified. A letter was read from Lord Brassey tendering his resignation as president, which was with regret accepted on the motion of Mr. H. N. Hamilton-Hoare. The Lord Mayor was elected president for the ensuing year. It was resolved, "That the sixteenth annual ladies' street collection be held on Saturday, July 13th next." On the motion of Mr. R. B. D. Acland, a "Weekly Collection Committee" was appointed for the purpose of promoting the "Penny Weekly Collection." Mr. Acland informed the board that a meeting had been held on the previous day at the Mansion House under the presidency of the Lord Mayor, when it was resolved that the Weekly Collection Committee should meet once a-month at the Mansion House. It was further resolved that a letter should be addressed from the Mansion House to all employers of labour and others whose co-operation is desirable.

